# PGMRA: a web server for (phenotype × genotype) many-to-many relation analysis in GWAS

**DOI:** 10.1093/nar/gkt496

**Published:** 2013-06-11

**Authors:** Javier Arnedo, Coral del Val, Gabriel Alejandro de Erausquin, Rocío Romero-Zaliz, Dragan Svrakic, Claude Robert Cloninger, Igor Zwir

**Affiliations:** ^1^Department of Computer Science and Artificial Intelligence, University of Granada, E-18071 Granada, Spain, ^2^Department of Psychiatry, Washington University School of Medicine, Campus Box 8230, 660 South Euclid Avenue, St. Louis, MO 63110, USA and ^3^Department of Psychiatry and Behavioral Neurosciences, University of South Florida, 3515 East Fletcher Avenue Tampa, FL 33613, USA

## Abstract

It has been proposed that single nucleotide polymorphisms (SNPs) discovered by genome-wide association studies (GWAS) account for only a small fraction of the genetic variation of complex traits in human population. The remaining unexplained variance or missing heritability is thought to be due to marginal effects of many loci with small effects and has eluded attempts to identify its sources. Combination of different studies appears to resolve in part this problem. However, neither individual GWAS nor meta-analytic combinations thereof are helpful for disclosing which genetic variants contribute to explain a particular phenotype. Here, we propose that most of the missing heritability is latent in the GWAS data, which conceals intermediate phenotypes. To uncover such latent information, we propose the PGMRA server that introduces phenomics—the full set of phenotype features of an individual—to identify SNP-set structures in a broader sense, i.e. causally cohesive genotype–phenotype relations. These relations are agnostically identified (without considering disease status of the subjects) and organized in an interpretable fashion. Then, by incorporating a posteriori the subject status within each relation, we can establish the risk surface of a disease in an unbiased mode. This approach complements—instead of replaces—current analysis methods. The server is publically available at http://phop.ugr.es/fenogeno.

## INTRODUCTION

Phenomics, defined as the acquisition of high-dimensional phenotype data on an organism-wide scale, has arisen as a possibility to address the ‘many-to-many’ relationships that are inherent in the phenotype and genotype domains of a disease (1). However, the interaction of phenomics with genomics in human diseases is usually precluded by a reduction of dimensionality of the phenotype features, which implies the elimination of their explanatory power. Although there is an increasing interest on identifying the key phenotype features associated with the genetic variants of a disease (2), there is a lack of methods devoted to extract the maximum information from these descriptors (1,3).

Phenotype–genotype relations have been often established using a modest numbers of single nucleotide polymorphisms (SNPs) associated with limited binary or discrete case-control phenotypes in genome-wide association studies (GWAS). These studies suffer from limited reproducibility, difficulties in finding causal SNPs because tagged SNPs are not necessarily causal, as well as in detecting multiple genetic sources (missing heritability), and inability to detect epistatic consequences (4–6). Therefore, recent approaches in genomics have focused on identifying functional sets of SNPs—instead of single SNPs—based on their proximity to particular genes or haplotype blocks to model the joint effect of multiple causal signals corresponding to multifaceted diseases (4). However, the sole identification of SNPs sets is not sufficient to explain the pleiotropic effects of the genetic variations in humans (1). Therefore, new methods are needed to identify, in an unbiased fashion, interpretable SNP-set structures in a broad sense, based on relations between sets of phenotype features coherently linked to SNP sets. To address this problem, we developed the PGMRA web server, which encodes methods that independently identify SNP sets and phenotype sets from GWAS data, and uncover optimal—instead of exhaustive—many-to-many phenotype–genotype relations among them. These methods also organize the uncovered coherent relations as networks in an interpretable topological fashion that, in turn, describe the risk surface of a disease.

PGMRA is, to our current knowledge, the only generic server that concurrently performs an exploratory and explanatory analysis of the phenomic and genomic domains of genome-wide data, pursuing the objective of uncovering the intermediate phenotypes (latent) concealed in the sample. The results obtained with PGMRA can be used as input for further analysis in other servers (4,7–11).

## SUMMARY OF FEATURES

PGMRA uses a generalized factorization method [see also relational clustering (12)], which combines factorization analysis, optimization research and conceptual clustering approaches to addresses the problem of discovering interesting clusters—substructures or concepts—defined in distinct domains (e.g. phenotype and genotype) and the associated problem of determining interesting relations between those clusters (13–15) (see Supplementary Methods).

Several characteristics distinguish PGMRA from other association approaches and avoid possible biases in the procedure: (i) the grouping strategy does not use previous knowledge about other studies (meta-analysis) or genomic features (pathway analysis) and does not consider the status of the subjects in the data set to identify either SNP or phenotype sets (i.e. unsupervised learning); (ii) subjects, SNPs and/or phenotype features can belong to more than one relation; (iii) SNPs within an SNP set can be located anywhere in the genome; (iv) the dimensionality of the phenotype features is not reduced (as would be the case with Principal Component Analysis or similar approaches) because, in phenomics, important features are *a priori* not known (1); (v) there is no predefined number of SNP sets and/or phenotype sets and/or relations among them (16); (vi) many-to-many relations among SNP and phenotype sets are identified in an unbiased fashion by using the probability of subject intersection (17), without considering subject’s disease status (e.g. cases, controls); and, most importantly, (vii) the risk of a disease is estimated in an unbiased fashion by incorporating *a posteriori* the subject status within each relation, weighing the frequency of each type of status (e.g. cases, relatives, controls) and mapping it into a predictive risk surface (18,19). Moreover, after the latter process, the statistical significance of interactions of SNPs associated with the disease can be estimated (4,7). In sum, PGMRA provides a quick snapshot of a single GWAS in an interpretable fashion.

## METHODOLOGY

**Figure 1.**
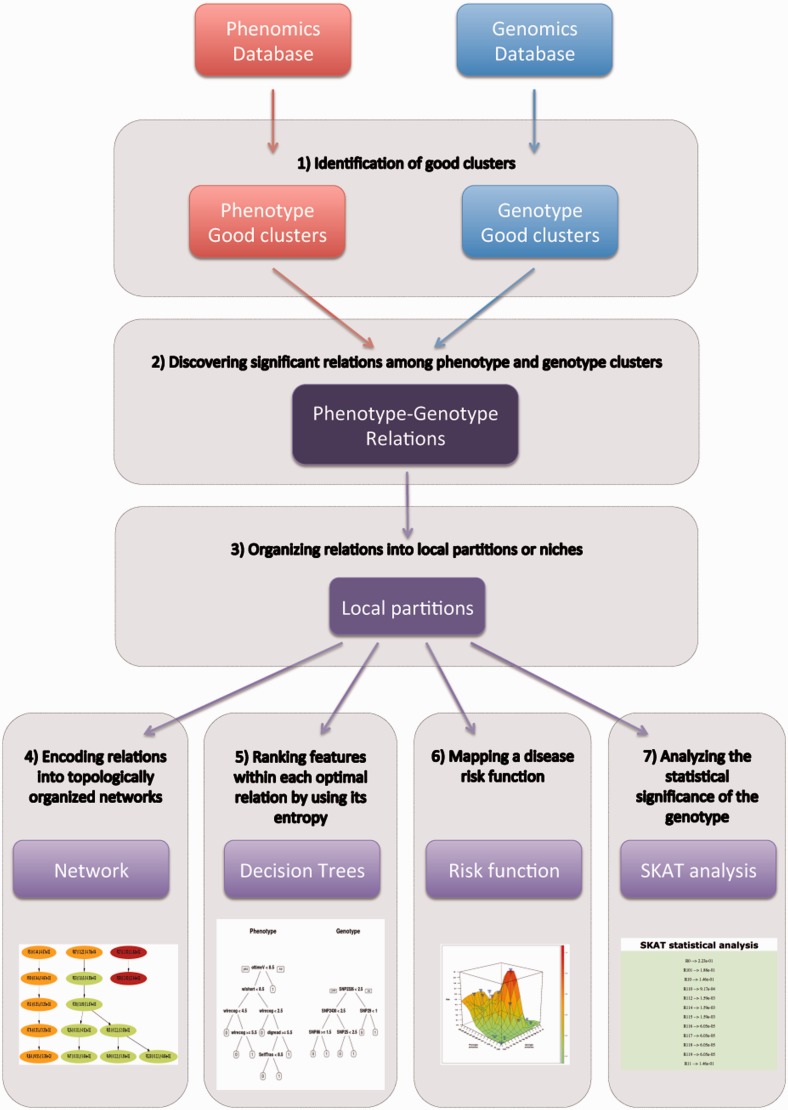
The workflow of the PGMRA web server. Processes involving phenotype and genotype clusters (biclusters) are indicated in red and blue, respectively, whereas procedures concerning phenotype–genotype relations are shown in violet.

Given a phenotype database (phenotype features × subjects) and a genotype database (SNPs × subjects), the approach followed by PMRGA is briefly described in the following seven steps (Figure 1, see Supplementary Methods):

1. *Identification of phenotype and genotype good clusters.* Factorization of the phenotype and genotype data is performed independently with the bioNMF method (20) or a novel version of a Nonnegative Matrix Factorization method (NMF) here proposed and termed Fuzzy NMF (FNMF). FNMF allows overlapping among sub-matrices and detection of outliers and is implemented as the default option (see Supplementary Figures S1–S4 for comparisons with bioNMF). Given a basic method, the generalized factorization method is applied recurrently to generate multiple clustering results using various initializations with different maximum numbers of clusters (e.g. from 2 to 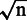, where *n* is the number of subjects) and thus avoids any pre-assumption about the ideal number of clusters. For each run within each genotype and phenotype domains, all clusters are selected composing a family of phenotype biclusters *P* = {*P_1_*, … ,*P_m_*}, as well as a separate family of genotype biclusters *G* = {*G_1_*, … ,*G_o_*}. Each family may include overlapped, partially redundant and different sizes of biclusters. Additionally, other basic biclustering methods have been implemented including Cheng and Church (21), FLexible Overlapped biClustering (FLOC) (22) and Factor Analysis for BIcluster Acquisition (FABIA) (23) (Supplementary Table S1), which are also extended by the generalized factorization method.
2. *Discovering relations among phenotype and genotype clusters*. The set of phenotype–genotype relations are identified by cross-correlating the phenotype and genotype biclusters, calculating the pairwise probability of intersection among them using the Hypergeometric statistics (PI_hyp_) (13,17) on the subject space [Supplementary Methods, Equation (4)]. [Permutations (24) are used to build an empirical random distribution of PI_hyp_ to estimate the upper non-random *P*-value of an identified relation.]
3. *Organizing relations into local partitions or niches.* To identify relations describing similar subjects, the method calculates the distance matrix *Ds* among all phenotype–genotype relations R*_i,j_* using the PI_hyp_ metric on the subjects space [Supplementary Methods, Equation (4)]. Then, the relations were clustered using *Ds* as an intra-clustering distance metric, where each resulting cluster constitutes a niche of relations describing similar subjects.
4. *Encoding relations into topologically organized networks.* To identify optimal and non-redundant relations, which may occur due to the repetitive application of the factorization method, the method calculates the distance matrix *Dp* among all phenotype–genotype relations R*_i,j_* within a niche by using the PI_hyp_ metric on the phenotype space [Supplementary Methods, Equation (4)]. Then, all relations with PI_hyp_ (R_i,j_, R_k,l_) < 10E-6 (i.e. sharing targeted subjects, phenotype and genotype features) are eliminated. The remained relations are hierarchically organized by inclusion of subjects using the Jaccard’s similarity metric (13,15) [Supplementary Methods, Equation (5)] and linked with arrows (>50% of inclusion). (Nested relations encode both sensitive and specific relations, both of which harbor distinct predictive power.)
5. *Ranking features within each optimal relation by using its entropy.* To identify the relevance of the features and their corresponding ranges of values that characterize each relation R_i,j_, the method labels each subject within and outside a relation with two different categorical (latent) classes. Then, for a given relation R*_i,j_*, the method calculates one decision tree (25) per domain, which ranks the utility of the features from top to bottom.
6. *Mapping a disease risk function.* To estimate the risk function of the network, the method incorporates *a posteriori* the status of the subjects composing each relation. Each relation is encoded into a 3-tuple (*X*,*Y*,*Z*), where *X* and *Y* correspond to the phenotype and genotype dimensions of the relation, respectively, and *Z* is the risk variable calculated by a weighted average of epidemiological risks of all subjects in the relation [Supplementary Methods, Equation (6)]. Relations are placed along the phenotype (*x*-axis) and genotype (*y*-axis) dimensions by clustering them using a phenotype and genotype distance matrices based on the PI_hyp_ metric. The 3-tuples are then interpolated and plotted.
7. *Analyzing the statistical significance of the genotype associated with the disease.* To calculate the significance of multiple SNPs within a SNP set belonging to a relation, we first run a labeling process that transforms an unsupervised relation into a supervised one. Then, we used the identity-by-state (IBS) kernel-machine method from the R-project package SKAT (4,7), which is a widely used approach to evaluate SNP sets, accounting for the multiple correction, as well as for the degrees of freedom of the test.

## IMPLEMENTATION

The PGMRA web server has been implemented using PHP, making use of a bash script that communicates with the different biclustering implementations, as well as with several post-processing Perl and R scripts. The biclustering methods are implemented in C, but they are used through their respective Perl or R wrappers. Several R-project packages are used in the PGMRA implementation: *pheatmap* for the heatmap graphs; *latticeExtra*, *akima*, *tgp*, *animation* and *plotrix* for the 3D risk graph; *rpart* and *rpart.plot* for the classification trees; *SKAT* for the statistical analysis of SNP sets; and *biclust*, *fabia* and *BicARE* for the Cheng&church, FABIA and FLOC biclustering methods, respectively (Supplementary Tables S1 and S2). The program *graphviz* was used for the network graph generation.

## PERFORMANCE

The time of execution can vary from few minutes to several hours depending on the size of input files and the selected parameters. The biclustering process is the most time-consuming step, but this time can be constrained by using one core for each K (e.g. maximum number of clusters K, 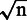). To test the performance of the method, we evaluated two GWAS of schizophrenia, which harbor different size and status of their subjects, as well as distinct phenotype features (see Supplementary Data Sets). These studies were termed ‘small’ (70 subjects × 8000 SNPs, which corresponds to the genotype and phenotype test files provided in PGMRA) and ‘big’ data sets (2500 subjects × 2700 SNPs), where the consuming time for a maximum of 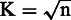 biclusters using the FNMF method was measured for different number of SNPs in each study (Supplementary Table S3).

We calculate the processing times in the PGMRA server, which is a 64-bit computer with 4core-2Ghz processors, as well as in the High Performance Computing hardware at Washington University (chpc.wustl.edu) by using a single core per K (HPC) and Message Passing Interface with 8 cores per K (HPC-MPI). The result reveals an improvement of ∼15 and ∼30% by using the HPC versus the PGMRA hardware in the big and small data sets, respectively. However, >90% of improvement is achieved by using the HPC-MPI option in both samples (Supplementary Table S3). Therefore, we provide on request an off-line MPI PGMRA additional service for big and time-consuming samples if the users do not want to wait the processing time in the open server.

The difference in performance of the distinct biclustering algorithms was also tested on the server by comparing the running time for the small data set for a fixed maximum number of biclusters K (Supplementary Table S4). Both bioNMF and FNMF methods allow different initializations *per* K (i.e. 40 by default) to avoid local minimum partitions, which eventually increases the time of processing. In contrast, the other methods do not explicitly allow such approach. Moreover, the former two classes of methods are qualitatively different, as they have different optimization fitness functions that eventually produce distinct results. For example, the Cheng&church method (21) tends to find homogeneous and crisp biclusters, whereas the bioNMF method (20) pursues the objective of identifying more general structures that facilitates the uncovering of inter-domain relations with other biclusters.

Additionally, the user can select to use the IBS kernel-machine test on each SNP sets found. The SKAT package is *per se* time consuming, and the user should take it into account when selecting this option. This processing time is partially increased because each SNP set/relation has to be independently submitted to SKAT, as they are not disjoint and cannot fill up a single input file.

## WEB INTERFACE

To start a job in the PGMRA server, the user has to click the tab ‘Send a job’ in the home page. This tab leads to the submission page where the user will be able to specify the input files and the desired parameters. Once all parameters are specified, the user can start the job by clicking the ‘send’ button. An email address specification is optional; nevertheless, it is encouraged owing to the long execution time required for large input files. If an email address is specified in the submission window, a job confirmation, as well as a notification is sent when the results are available. Moreover, we provide a list constantly updated actions (e.g. pre-processing, biclustering discovery, etc.) that reflect the progress of the submitted job. PGMRA has detailed tutorial available along with real GWAS example test files. The tutorial explains which parameters can be tuned and between which ranges they can be modified. Default settings should be adopted for beginners (Supplementary Table S2).

### Input

Three files compose the PGMRA input: the phenotype (phenotype features × subjects) and genotype (SNPs × subjects) files, which are matrices in a tab-separated format, and the subject status file (e.g. case or control). The genotype file contains information about the genotype variables of each subject in the study but in a numeric form. The SNP alleles are represented by a combination of numeric values (i.e. 1 = AA, 2 = AB, 3 = BB and 0 = missing value). The status file contains the conversion of the status classification into numeric values (i.e. 1 = case, 2 = relative and 3 = control).

The genotype and subject status files can be also generated from the standard files used by the Plink software by running the script plink2pgmra.pl provided in the PGMRA website. To do that, the Plink program has to be run using the binary files obtained from the GWAS analysis (*.bed, *.bim, *.fam) as inputs and the parameters –recode12 and –transpose. This process generates two output text files (*.tped, *.tfam) that constitute the inputs of the plink2pgmra.pl script to obtain the genotype and status files. The phenotype file, which is a matrix where the features are the rows and the subjects the columns, has to be provided by the user. The values should be normalized between minimum and maximum values of each feature. The current example encoding a GWAS of schizophrenia (see Supplementary Data Sets) has 8007 SNPs; however, we encourage the use of a pre-selection of SNPs with relaxed *P*-values by using the Plink software for larger samples (26). Finally, if the user selects the statistical analysis of the SNP sets, PGMRA automatically converts the input genotype and status files into Plink binary files, which are required input by the SKAT method.

### Parameters

The PGMRA server requires setting up two types of parameters related to the basic biclustering methods and to the generalized factorization method. The default basic biclustering method is FNMF, and the alternative biclustering methods are only recommended for users experienced in clustering aware of their own characteristics (Supplementary Tables S1 and S2, see Supplementary Methods). The corresponding list of running parameters for each method is also reported (Supplementary Table S2). The new basic biclustering FNMF method—based on the bioNMF method (20)—introduces the levels of fuzziness as a new parameter (0.5 default value), which allows generating more flexible biclusters (higher values) or more cohesive ones (lower values, see Supplementary Methods).

Second, because all basic methods are run as sub-processes of the generalized factorization method, the minimum and maximum number of clusters (biclusters) where the basic methods are repetitively run have to be specified (e.g. between 2 and and 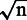 by default). This method also requires the specification of the minimum level of coincidence between a phenotype and a genotype biclusters, which determines the quality of the relation between them. Because this matching is calculated by the probability of intersection between two clusters using the PI_hyp_, low values used as a threshold generate less but more cohesive relations. In contrast, the use of high threshold will admit more relaxed relations—eventually all relations (i.e. *P*-value = 1)—to the hierarchically organized network and, thus, to integrate the risk surface of the disease. We established *P*-value < 0.05 as the default threshold.

### Output

The output page shows a snapshot of the analysed GWAS in seven graphical output sections. First, a summary with the name of the input data, method and parameter selected is displayed in the result page, making possible the tracking of results. Then, a statistical summary of the run exhibits information about the total number of identified relations, as well as the average number of subject, phenotype features and SNPs per relation. Second, the network composed of significant relations are hierarchically organized when describing approximately the same target subjects from different phenotype and/or genotype features. Remarkably, the network is organized without information about the subject status (i.e. unsupervised learning), which is incorporated *a posteriori* into each relation to calculate its risk. Each node contains the relation name, its associated risk and the degree of matching between the phenotype and genotype biclusters that originated the relation (PI_hyp_). Relations are color coded by their corresponding risk values. Third, the risk barchart output section shows the distribution of the risk among all relations. Fourth, the relation-mapping output section provides two heatmaps, one phenotype and another genotype, where the features (columns) characterizing each relation (rows) are highlighted. Fifth, two classification trees—one phenotype and another genotype—represent each relation, where the features are ordered (top to bottom) by their importance as calculated by the entropy. Sixth, the risk surface of the disease is plotted and shown in a 3D dynamic graph. And, seventh, a list of relations and their corresponding *P*-values resulting from the application of the IBS kernel-machine method from the R-project package SKAT is shown. All results are provided in HTML format for direct visual inspection in browser. All images shown in each window can be downloaded, and the subjacent data can be accessed as text files through the provided links. Finally, each output also summarizes the input file names, as well as the method and parameters selected.

## DISCUSSION

GWAS have emerged as popular tools for identifying genetic variants associated with the risk of a disease. However, the effect of this approach was limited in complex diseases like schizophrenia, where >1000 genes are implicated. Therefore, we developed the PGMRA web server, which encodes a conceptual framework to dissect a GWAS into coherent many-to-many phenotype–genotype relations that encode multifaceted data interactions. These relations, organized as a network, provide a snapshot of what effectively is in a single GWAS experiment—as opposed to what could be when adding external information (meta- or pathway analysis)—in an interpretable graphical fashion.

Three novel algorithms have been incorporated and combined in the PGMRA server: the basic biclustering FNMF method, which extends the bioNMF method allowing overlapping and outlier detection of biclusters; a generalized factorization method, which fuse inter-domain biclusters into relations in an efficient unsupervised fashion; and a data-labeling strategy, which transforms unsupervised latent relations into supervised structures by incorporating their risk in an unbiased manner (see Supplementary Methods, G. A. de Erausquin *et al.*, unpublished data). The latter strategy allows calculating the ranking of the features within a relation as measured by their entropy, as well as to plot a predictive risk surface of the subjacent disease.

The PGMRA server has been successfully applied separately to both large- and small-scale schizophrenia data sets (see Supplementary Data Sets). These GWAS were partly carried out in Washington University School of Medicine (19,27,28), harboring typical case-control phenotypes or extended phenotypes, where relatives at risk were also considered. The obtained results empirically demonstrated that genetic risk of schizophrenia occurs along a continuum and that the phenotype–genotype relations are capable of identifying the corresponding intermediate phenotypes shared by patients and their first-degree relatives (see Supplementary Data Sets, G. A. de Erausquin *et al.*, unpublished data). Moreover, these relations were independently curated and cataloged by experts (psychiatrist-based nosological framework represented by the Diagnostic and Statistical Manual of Mental Disorders (DSM-IV)), who determined that they distinguish positive from negative symptoms of schizophrenia, which, in turn, dissect the disease into paranoid and non-paranoid classes. This suggests that the differential genetic basis of the relations may dictate differential and personalized treatments of the disease (see Supplementary Data Sets, J. Arnedo *et al.*, unpublished data).

It is essential to note that two types of statistical evaluations (*P*-values) are reported, namely, one for the probability of an intersection (PI_hyp_) between a SNP set and a phenotype feature set (i.e. the statistical significance of ‘relations’) evaluated by the Hypergeometric statistics and compared with an empirical random distribution of PI_hyp_ used to estimate the upper non-random *P*-value of an identified relation. Another evaluation was performed for the probability of an SNP set being associated with schizophrenia (i.e. the genome-wide association significance) calculated by the logistic kernel-machine statistics.

The uncovered phenotype–genotype relations at risk using the NMF method in the big data set provided significant values as evaluated by the PI_hyp_: 1E-05 < *P* < 7E-13. Moreover, the set of SNPs included in most of the significant relations were more statistically significant (e.g. *P* < E-05 or even *P* < E-11) than the best individual SNPs included in the corresponding sets when were evaluated by the SKAT method (4) (i.e. many orders of magnitude lower, Supplementary Table S5). Similarly, the phenotype–genotype relations from the small data set obtained the following PI_hyp_ values by using the bioNMF method: 1E-05 < *P* < 3E-10. Likewise, the big data set, the SNP sets evaluated by the SKAT method displayed better values than the individual SNPs either with the bioNMF (1E-05 < *P* < 2E-08) or with the FNMF (*P* < 1E-05, up to *P* < 2E-08) methods (Supplementary Table S5). This approach narrows down the problem of multiple comparisons exhibited by typical GWAS analysis (4,29), as expected.

Notably, many of these relations have SNPs located all across the genome—instead of within a gene or haplotype (4)—that are associated with many genes previously related to schizophrenia, as well as with novel ones (e.g. non-coding RNA genes) or to other regulatory features. The found genes have been primarily associated to mental, brain and nervous system disorders as cataloged by the NextBio (www.nextbio.com, scores > 80) and Ingenuity® Pathways Analysis (IPA) (www.ingenuity.com, *P* < 5.2E-05) servers (J. Arnedo *et al.*, unpublished data). Moreover, many of the identified genes linked to hypodopaminergic phenotypes form a highly interconnected network that can be parceled out to just a few major functional pathways, encoding almost all essential component in the neuronal cell adhesion (IPA, *P* < 3E-05) or in the small GTPase or signaling pathway (IPA, *P* < 2E-04) (E. G. *et al.*, unpublished data). Other mapped genomic regions overlap newly characterized long intergenic non-coding RNAs—coincident with an annotated CNV—including the lincRNAs AC068490.2 and AC096570.2, which expression may cause alterations of the normal brain development (J. Arnedo *et al.*, unpublished data). Overall, these cohesive relations show that the phenotype–genotype relations are not just a computational artifact but encode a profound biomedical meaning.

The results obtained with PGMRA can easily interact with other servers, programs and databases. For example, once identified the SNP-subsets in each optimal relation, they can be easily used for downstream analysis such as (i) functional annotation: Haploreg webservice (8), ENCODE (genome.ucsc.edu/ENCODE) and ENSEMBL database (www.ensembl.org) (11); (ii) network and pathway analysis: DAVID (34), Genecards (35), Prolinks Database 2.0 (36), IPA (www.ingenuity.com), ICSNPathway (10); (iii) analysis of diseases and drugs related to the affected genes: Nextbio webservice (9) ‘Disease Atlas’ and ‘Pharmaco Atlas’.

We think that the server here presented can help to understand complex diseases landscapes in relation to genotypic variation, bringing some light into multiple SNPs’ cross-effects. The development of the server presented here has been user driven from the beginning. Its functionality is continually being updated and extended in response to requests and suggestions emerging from our core users.

## SUPPLEMENTARY DATA

Supplementary Data are available at NAR Online: Supplementary Tables 1–5, Supplementary Figures 1–5, Supplementary Methods, Supplementary Data Sets and Supplementary References [30–33,37–40].
